# Family planning under sociocultural influence: evaluating knowledge, access, and utilization in Al Baha, Saudi Arabia

**DOI:** 10.3389/fgwh.2026.1770117

**Published:** 2026-03-20

**Authors:** Mohammad A. Albanghali, Uthman Albakri, Hayat A. Alghamdi, Weiam A. Al Fahad, Mead M. Alzahrani, Haneen A. Alamri, Batol M. Albanghali, Norah A. Alghamdi, Amer A. Alshehri, Saleh Alghamdi, Dina Marghani, Soltan J. Algamdi, Mohammed A. Shanawaz, Basim A. Othman

**Affiliations:** 1 Department of Public Health, Faculty of Applied Medical Sciences, Al-Baha University, Al Baha, Saudi Arabia; 2 Faculty of Medicine, Al-Baha University, Al Baha, Saudi Arabia; 3 Faculty of Pharmacy, Al-Baha University, Al Baha, Saudi Arabia; 4 Department of Clinical Pharmacy, Faculty of Pharmacy, Al-Baha University, Al Baha, Saudi Arabia; 5 Department of Clinical Laboratory Science, Faculty of Applied Medical Science, Taibah University, Madina, Saudi Arabia

**Keywords:** Al-Baha province, contraception, family planning, health services accessibility, reproductive health services, Saudi Arabia, socioeconomic factors

## Abstract

**Background:**

Unmet need for contraception remains a persistent global reproductive health challenge. In Saudi Arabia, national estimates indicate moderate contraceptive uptake; however, region-specific data on awareness, accessibility, and utilization of family planning (FP) services remain limited, particularly in semi-rural settings such as Al-Baha.

**Objective:**

To evaluate awareness, access, and utilization of family planning and reproductive health services and to identify perceived barriers and sociodemographic determinants among adult women in Al-Baha, Saudi Arabia.

**Methods:**

A population-based cross-sectional study was conducted using a 23-item online questionnaire administered to female residents aged ≥18 years between December 2024 and February 2025 through convenience sampling. Descriptive statistics summarized participant characteristics and FP-related variables. Associations between categorical variables were assessed using chi-square (*χ*²) tests. Univariable and multivariable logistic regression analyses were performed to estimate crude and adjusted odds ratios (ORs) with 95% confidence intervals (CIs). Statistical significance was set at a two-sided *p* < 0.05. Ethical approval was obtained from the Deanship of Scientific Research, Al-Baha University (Approval No. 46110701–20241117), and electronic informed consent was secured prior to participation.

**Results:**

A total of 892 women participated (mean age 38 ± 10.5 years). Awareness of FP initiatives was reported by 24%, and 18% had previously received FP consultation/services. Although 94% reported geographic access to a healthcare facility within 30 min, routine reproductive health service utilization remained limited, with 53% reporting infrequent visits. Current contraceptive use was reported by 42%, predominantly oral contraceptive pills (15%) and intrauterine methods (9%). Overall, 32% reported at least one barrier to contraception access, with “other barriers” (14%) and limited availability (11%) cited most frequently. Several outcomes demonstrated significant sociodemographic variation (*p* < 0.05). In multivariable analysis, age >30 years independently predicted current contraceptive use (aOR 3.97; 95% CI 2.64–5.98; *p* < 0.001).

**Conclusion:**

Despite reported geographic accessibility, awareness and counselling uptake remain suboptimal in Al-Baha. Targeted, culturally responsive primary care-based interventions and strengthened service availability are warranted to improve informed reproductive health decision-making.

## 1 Background

Family planning and reproductive health are fundamental to maternal and child wellbeing, gender equity, and sustainable socioeconomic development. Effective reproductive health strategies empower individuals to make informed decisions regarding fertility, contraception, and maternal care, ultimately reducing maternal and infant morbidity and mortality (1, 2). Globally, fertility rates have declined substantially over the past several decades due to increased access to contraception, improvements in women's education, urbanization, and economic development. Despite this global transition, significant disparities in reproductive health access and family planning utilization persist, particularly in rural and underserved populations, contributing to unmet reproductive health needs worldwide (3–7). In Saudi Arabia, fertility patterns have undergone a profound transformation over the past decades, influenced by evolving sociocultural norms, economic shifts, and healthcare advancements (8). Historically, Saudi women had an average of seven children in the 1970s, a figure that has declined to approximately 2.3 births per woman. This decline is attributed to expanded educational and career opportunities for women, increased economic participation, and government led public health initiatives that have prioritized reproductive health awareness and accessibility (9, 10).

Despite these national advancements, regional disparities persist, particularly in rural and less urbanized areas such as Al-Baha and its surrounding provinces. Access to family planning resources, reproductive health education, and maternal healthcare services varies considerably across different regions, with rural communities often experiencing limited healthcare infrastructure and greater sociocultural barriers (5, 6, 11). Understanding these disparities is critical for developing targeted, evidence based interventions that enhance reproductive health services and address barriers to family planning utilization. In alignment with Vision 2030, Saudi Arabia has made significant progress in improving women's healthcare access, economic independence, and reproductive health services. Policies promoting women's empowerment and workforce participation have facilitated shifts in reproductive behaviors, leading to increased adoption of family planning practices (12–14). However, despite these advancements, sociocultural and economic influences remain strong determinants of fertility decisions in many communities. Traditional family structures in certain regions continue to favor larger households, influenced by cultural expectations and intergenerational norms. Economic factors further contribute to shifting fertility patterns. The rising cost of living, including housing, childcare, and education expenses, has led many families to reconsider their reproductive choices, often prioritizing financial stability over larger family sizes. Additionally, environmental and lifestyle factors, such as dietary shifts, pollution, and increasing rates of metabolic disorders like obesity and diabetes, have been linked to declining fertility rates, complicating reproductive health outcomes (15–17). These complex interrelated factors highlight the need for region specific reproductive health policies that address both economic constraints and public health concerns. Al Baha represents a unique case where traditional sociocultural values intersect with contemporary economic realities. While urban centers have adapted to modern family planning approaches, many rural communities continue to uphold conservative reproductive norms, viewing large families as economically advantageous and socially desirable. In agricultural economies, children often contribute to household labor and economic productivity, reinforcing preferences for larger family sizes (18, 19). Religious and cultural beliefs also play a pivotal role in shaping attitudes toward contraception and assisted reproductive technologies (ART). While national policies promote contraceptive access and reproductive health education, perceptions of ART and contraceptive use vary significantly across different social groups (20, 21). In Al Baha, cultural and religious beliefs may influence fertility preferences and attitudes toward family planning; however, the extent to which these beliefs shape reproductive health behaviors and service utilization remains insufficiently explored. Examining this relationship is important for understanding family planning patterns in the region (18, 20–22).

In several communities, family planning is often viewed as conflicting with religious or traditional beliefs, which can hinder the adoption of modern contraceptive methods. Such perceptions emphasize the need for culturally sensitive public health interventions that honor local values while promoting evidence based reproductive health practices (21). In rural areas of Al Baha, access to reproductive health services remains limited due to inadequate infrastructure, provider shortages, and the absence of specialized care such as contraceptive counseling, fertility treatment, and maternal health support (23). These limitations are further compounded by sociocultural stigma, which discourages open discussion and utilization of reproductive health services. Many individuals avoid consultations out of fear of social judgment or violating cultural norms, resulting in unmet family planning needs and higher fertility rates. Addressing these multifaceted barriers requires tailored, community driven strategies, investment in healthcare infrastructure, and culturally informed education initiatives. Although national level studies have documented fertility decline and reproductive health improvements in Saudi Arabia, there remains a lack of region-specific research examining the structural, economic, and sociocultural determinants of reproductive behavior in rural areas such as Al Baha. This gap limits the development of targeted, culturally appropriate reproductive health interventions tailored to local needs.

This study seeks to identify and analyze the structural, economic, and cultural factors shaping reproductive behaviors in Al Baha, offering evidence-based recommendations for improving reproductive health policy and service delivery in the region. This study examines community awareness, accessibility, and utilization of family planning and reproductive health services in Al Baha, analyzing sociocultural, economic, and environmental determinants of reproductive decision making. It evaluates participation in public health initiatives, the impact of awareness campaigns, and barriers to contraception and assisted reproductive technologies. Findings will inform evidence-based strategies to enhance reproductive health education and service accessibility.

## 2 Materials and methods

### 2.1 Study design

The study followed cross-sectional design to assess awareness, accessibility, and utilization of family planning and reproductive health services among female residents of Al Baha, Saudi Arabia. A cross-sectional approach was selected as it is well suited for examining population level patterns, attitudes, and associations between demographic characteristics and reproductive health behaviours.

### 2.2 Sample collection and survey tool

To gain a deeper understanding of family planning and reproductive health in Al Baha, data were collected using an online self-administered questionnaire distributed between December 15, 2024, and February 28, 2025. The survey was shared via email and WhatsApp, allowing for broad community participation while ensuring convenience and accessibility. Participation was voluntary, and all responses were collected anonymously to uphold confidentiality and encourage honest input. Online distribution was selected to facilitate wide community reach and standardized data collection. Nevertheless, as participation required internet access and voluntary response, some degree of selection bias cannot be excluded.

The questionnaire was designed to capture the diverse factors shaping reproductive health behaviours and consisted of 23-items covering Reproductive Health Awareness and Knowledge, Service Utilization and Engagement, Access and Service Availability/Barriers, Family Planning Behaviour and Fertility Preferences, Sociocultural and Economic Determinants, Health and Lifestyle. Before dissemination, the questionnaire was validated by a panel of experts in public health and reproductive medicine to ensure clarity, relevance, and reliability. Responses were collected and analysed electronically, minimizing errors and biases while enabling a structured, data driven assessment of family planning and reproductive health dynamics in Al Baha.

### 2.3 Statistical analysis

Data were analysed using IBM SPSS Statistics (Version 21.0; IBM Corp., Armonk, NY, USA). Descriptive statistics were used to summarise participants' characteristics and family-planning–related measures; continuous variables are presented as mean ± standard deviation (SD) and categorical variables as frequencies and percentages. Associations between categorical variables were assessed using the chi-square (*χ*²) test. Univariable logistic regression was conducted to estimate crude odds ratios (uORs) with 95% confidence intervals (CIs), followed by multivariable logistic regression to obtain adjusted odds ratios (aORs) with 95% CIs. A two-sided *p*-value < 0.05 was considered statistically significant, and results were reported in tables and figures.

### 2.4 Ethical considerations

Participation was voluntary, and electronic informed consent was obtained prior to questionnaire completion after providing participants with information on the study purpose. No personally identifiable information was collected; responses were anonymous and handled confidentially, with access limited to the research team. Results are reported in aggregate to minimise any risk of identification. The study was approved by the Deanship of Scientific Research, Al Baha University (Approval No. 46110701–20241117).

## 3 Results

### 3.1 Participants' characteristics

The study included 892 participants with a mean age of 38 ± 10.5 years. The majority (52%) were aged 30–46 years, while 76% were married. Among participants (Table 1), 52% were aged 30–46 years, while 24% were 18–29 years and 24% were 47–64 years. Most respondents were overweight (54.3%) or normal weight (35%), with 9.6% underweight and 1.1% obese. Residence was mainly village based (57.4%), followed by city (41.5%) and hamlet (1.1%). Employment status showed 50.9% employed, 21.7% housewives, 17% unemployed, and 10.3% students. Monthly household income was most commonly 5,000–10,000 SAR (36.1%), followed by 10,001–15,000 SAR (23.5%) and 15,001–20,000 SAR (19.5%), with 11.7% reporting <5,000 SAR and 9.2% > 20,000 SAR. Most participants were married (76%), and education was predominantly graduate level (80%).

**Table 1:** Sociodemographic characteristics and association with number of children among respondents in Al-Baha, Saudi Arabia (*N* = 892).

Variable	N (%)	Number of children				*p*-value	Cramér’s V
		0	1–3	4–5	≥ 6		
		184 (20.6%)	298 (33.4%)	314 (35.2%)	96 (10.8%)		
Age (years)							
18–29	210 (24%)	162 (77.1%)	48 (22.9%)	0 (0.0%)	0 (0.0%)	<0.001	0.586
30–46	466 (52%)	12 (2.6%)	206 (44.2%)	202 (43.3%)	46 (9.9%)		
47–64	216 (24%)	10 (4.6%)	44 (20.4%)	112 (51.9%)	50 (23.1%)		
BMI							
Underweight	86 (9.6%)	52 (28.3%)	24 (8.1%)	8 (2.5%)	2 (2.1%)	<0.001	0.257
Normal	312 (35%)	86 (46.7%)	114 (38.3%)	92 (29.3%)	20 (20.8%)		
Overweight	484 (54.3%)	46 (25.0%)	160 (53.7%)	210 (66.9%)	68 (70.8%)		
Obese	10 (1.1%)	0 (0.0%)	0 (0.0%)	4 (1.3%)	6 (6.2%)		
Type of City							
City	370 (41.5%)	76 (41.3%)	114 (38.3%)	142 (45.2%)	38 (39.6%)	0.242	0.063
Hamlet	10 (1.1%)	2 (1.1%)	8 (2.7%)	0 (0.0%)	0 (0.0%)		
Village	512 (57.4%)	106 (57.6%)	176 (59.1%)	172 (54.8%)	58 (60.4%)		
Employment status							
Employed	454 (50.9%)	28 (15.2%)	154 (51.7%)	208 (66.2%)	64 (66.7%)	<0.001	0.365
Housewife	194 (21.7%)	6 (3.3%)	88 (29.5%)	80 (25.5%)	20 (20.8%)		
Student	92 (10.3%)	86 (46.7%)	4 (1.3%)	2 (0.6%)	0 (0.0%)		
Unemployed	152 (17%)	64 (34.8%)	52 (17.4%)	24 (7.6%)	12 (12.5%)		
Household’s Monthly Income (SAR)							
Less than 5,000	104 (11.7%)	34 (18.5%)	32 (10.7%)	30 (9.6%)	8 (8.3%)	0.007	0.069
5,000–10,000	322 (36.1%)	60 (32.6%)	122 (40.9%)	114 (36.3%)	26 (27.1%)		
10,001–15,000	210 (23.5%)	46 (25.0%)	70 (23.5%)	60 (19.1%)	34 (35.4%)		
15,001–20,000	174 (19.5%)	30 (16.3%)	52 (17.4%)	72 (22.9%)	20 (20.8%)		
> 20,000	82 (9.2%)	14 (7.6%)	22 (7.4%)	38 (12.1%)	8 (8.3%)		
Marital status							
Married	678 (76%)	24 (13.0%)	272 (91.3%)	294 (93.6%)	88 (91.7%)	<0.001	0.449
Divorced	40 (4.5%)	2 (1.1%)	24 (8.1%)	10 (3.2%)	4 (4.2%)		
Single	156 (17.5%)	156 (84.8%)	0 (0.0%)	0 (0.0%)	0 (0.0%)		
Widowed	18 (2%)	2 (1.1%)	2 (0.7%)	10 (3.2%)	4 (4.2%)		
Education level							
Primary	8 (0.9%)	0 (0.0%)	0 (0.0%)	4 (1.3%)	4 (4.2%)	<0.001	0.128
Intermediate	18 (2%)	0 (0.0%)	6 (2.0%)	4 (1.3%)	8 (8.3%)		
Secondary	118 (13%)	28 (15.2%)	22 (7.4%)	50 (15.9%)	18 (18.8%)		
Graduate	714 (80%)	152 (82.6%)	258 (86.6%)	238 (75.8%)	66 (68.8%)		
Postgraduate	34 (4%)	4 (2.2%)	12 (4.0%)	18 (5.7%)	0 (0.0%)		

Data are presented as *n* (%), where percentages within the “Number of children” columns represent column percentages (i.e., % within each children category). *P*-values were obtained using the Pearson chi-square test of independence. Cramér’s V is reported as a measure of effect size for the chi-square association (0 = no association; higher values indicate stronger association). Statistical significance was set at *p* < 0.05. Children categories were defined as 0, 1–3, 4–5, and ≥6. BMI, body mass index; SAR, Saudi Riyal.

Patterns in number of children differed across several characteristics (Table 2). The number of children was associated with age (*p* < 0.001; Cramér's V = 0.586), with participants aged 18–29 years mostly reporting no children, whereas older age groups more often reported 4–5 children or ≥6. Number of children also varied by marital status (*p* < 0.001; V = 0.449) and employment status (*p* < 0.001; V = 0.365), with single participants and students predominantly reporting no children, while married, employed, and housewife groups more frequently reported higher parity. Associations were also observed for BMI (*p* < 0.001; V = 0.257), household income (*p* = 0.007; V = 0.069), and education level (*p* < 0.001; V = 0.128), whereas type of city was not associated with Number of children (*p* = 0.242; V = 0.063).

**Table 2:** Participant responses across domains of awareness/knowledge, service utilisation, access/barriers, family planning behaviour, sociocultural determinants, and health/lifestyle.

Domains	Items	*N* (%)
Awareness and Knowledge	Awareness of family planning public health initiatives	
	Yes	210 (23.5%)
	Knowledge of age-related fertility decline	
	Extensive knowledge	314 (35.2%)
	Moderate knowledge	456 (51.1%)
	Not knowledgeable	122 (13.7%)
	Primary source of reproductive health information	
	Friends	78 (8.7%)
	Healthcare	168 (18.8%)
	Media	456 (51.1%)
	Other sources	190 (21.3%)
Service Utilization and Engagement	Receipt of family planning counselling/services	
	Yes	160 (18.0%)
	Frequency of reproductive health service visits	
	Frequently (Every 1–3 months)	174 (19.5%)
	Occasionally (Every 6 months)	246 (27.6%)
	Rarely (Once a year or less)	472 (52.9%)
	Participation in community health events	
	Repeatedly	8 (0.9%)
	Sometimes	122 (13.7%)
	Rarely	228 (25.6%)
	Never	534 (59.9%)
	Perceived benefit from reproductive health awareness/education	
	Excellent	88 (9.9%)
	Good	256 (28.7%)
	Acceptable	212 (23.8%)
	Weak	336 (37.7%)
Access and Service Availability/Barriers	Perceived availability of family planning methods locally	
	Yes	578 (65.0%)
	Difficulty accessing contraception locally	
	High cost	34 (3.8%)
	Lack of awareness about available options	30 (3.4%)
	Limited availability	96 (10.8%)
	Social restrictions	6 (0.7%)
	Other	122 (13.7%)
	None	604 (67.7%)
	Access to a healthcare center within 30 min	
	Yes	842 (94.0%)
Family Planning Behavior and Fertility Preferences	Current contraceptive use	
	Condoms	26 (2.9%)
	Injections	10 (1.1%)
	Intrauterine device (IUD)	80 (9.0%)
	Natural methods	60 (6.7%)
	Oral Contraceptive Pills	130 (14.6%)
	Other	64 (7.2%)
	None	522 (58.5%)
	Main determinants of desired family size	
	Cultural expectations	18 (2.0%)
	Financial reasons	82 (9.2%)
	Personal desire	550 (61.7%)
	Other reasons	242 (27.1%)
	Preferred number of children	
	1–3	212 (24.0%)
	4–5	596 (67.0%)
	6 or more	84 (9.0%)
Sociocultural and Economic Determinants	Household income influences family-size decisions	
	Yes	512 (57.0%)
	Children viewed as financial support	
	Yes	320 (36.0%)
	Impact of financial constraints on having more children	
	Very high effect	254 (28.5%)
	Moderate effect	252 (28.3%)
	No effect	386 (43.3%)
	Community norms influence family-size decisions	
	Yes	262 (29.0%)
	Family/cultural pressure to have more children	
	Yes	222 (25.0%)
	Cultural acceptability of family planning	
	Accepted	542 (61.0%)
	Neutral	314 (35.0%)
	Not accepted	36 (4.0%)
Health and Lifestyle	Reproductive health conditions (e.g., diabetes, PCOS)	
	Yes	194 (22.0%)
	Diet quality in relation to reproductive health	
	Excellent	184 (21.0%)
	Acceptable	216 (24.0%)
	Good	432 (48.0%)
	Weak	60 (7.0%)
	Physical activity level	
	Daily	90 (10.1%)
	Several times a week	290 (32.5%)
	Rarely	412 (46.2%)
	I never exercise	100 (11.2%)
	Smoking or Use Tobacco Products?	
	Yes	10 (1.0%)

Questionnaire items are organised into six domains: awareness and knowledge; service utilisation and engagement; access and service availability/barriers; family planning behaviour and fertility preferences; sociocultural and economic determinants; and health and lifestyle.

### 3.2 Awareness and knowledge related to family planning

Overall awareness of public health initiatives related to family planning in Al-Baha was limited, with 210 (23.5%) of participants reporting awareness and 682 (76.5%) reporting no awareness. Familiarity with age related fertility decline was generally moderate to high: 314 (35.2%) reported extensive knowledge, 456 (51.1%) reported moderate knowledge, and 122 (13.7%) reported being not knowledgeable. Media was the most reported source of reproductive health information (51.1%), followed by healthcare sources (18.8%), other sources (21.3%), and friends (8.7%) (Table 2).

### 3.3 Service utilisation and engagement in reproductive health activities

Engagement with formal family planning services was limited: only 18% reported ever receiving family planning consultations or services, while 82% had not. Routine utilisation of reproductive health services was also low, with just 19.5% reporting frequent visits (every 1–3 months); most participants reported visiting occasionally (27.6%) or rarely (52.9%, once a year or less). Participation in community health events was uncommon. Nearly 59.9% reported never participating, 25.6% participated rarely, and 13.7% sometimes participated; only 0.9% reported repeated participation. Perceived benefit from awareness campaigns and health education programmes also appeared modest: 37.7% rated the benefit as weak, compared with 28.7% good, 23.8% acceptable, and 9.9% excellent (Table 2).

### 3.4 Availability, access, and barriers to contraceptive services

Perceived availability of family planning tools was relatively high, with 65.0% reporting that tools were available in their area. Geographic access to services also appeared favourable, with 94.0% reporting they could reach a healthcare centre within 30 min. Despite this, a notable minority reported barriers to accessing contraception. Overall, 32.3% reported experiencing at least one challenge, most commonly, limited availability (10.8%) and other barriers (13.7%), followed by high cost (3.8%) and lack of awareness about available options (3.4%). Social restrictions were least commonly reported (0.7%). The remaining 67.7% reported no challenges (Table 2).

### 3.5 Family planning behaviour and fertility preferences

More than half of participants reported not currently using contraception (58.5%). Among those reporting contraceptive use, oral contraceptive pills were the most frequently reported method (14.6%), followed by intrauterine methods (9.0%), other methods (7.2%), natural methods (6.7%), condoms (2.9%), and injections (1.1%). When asked about drivers of decisions to have more or fewer children, personal desire was the most reported reason (61.7%), followed by other reasons (27.1%), financial reasons (9.2%), and cultural expectations (2.0%). Regarding fertility preferences, most participants identified 4–5 as the ideal number of children (67.0%), while 24.0% selected 1–3, and 9.0% selected 6 or more (Table 2).

### 3.6 Sociocultural and economic influences on family size decisions

Economic considerations were commonly reported in relation to family size decisions. More than half of participants indicated that household income affects their decisions about family size (57.0%). However, only 36.0% reported that children are considered a source of financial support in their family, while 64.0% did not. Financial constraints were not uniformly experienced: 43.3% reported no effect, whereas 28.5% reported a very high effect and 28.3% a moderate effect on decisions about having more children. Sociocultural influences were reported less frequently. Most participants indicated that community values do not influence their decisions about family size (71.0%), and 75.0% reported no cultural or family pressure to have more children. When asked about the role of family planning within cultural values, 61.0% considered it accepted, 35.0% were neutral, and 4.0% reported it was not accepted (Table 2).

### 3.7 Health and lifestyle characteristics relevant to reproductive health

Self-reported reproductive health issues were reported by 22.0% of participants, while 78.0% reported none. Perceived diet quality was most frequently rated as good (48.0%), followed by acceptable (24.0%) and excellent (21.0%); a smaller proportion rated diet quality as weak (7.0%). Physical activity levels were generally low: 46.2% reported engaging rarely, 32.5% several times per week, 10.1% daily, and 11.2% reported never exercising. Tobacco use was uncommon, with 1.0% reporting smoking or tobacco use and 99% reporting none (Table 2).

### 3.8 Associations of key family planning outcomes with sociodemographic factors

Across the eight key outcomes, several showed sociodemographic variation (Table 3). Awareness of family planning (FP) initiatives differed by age (*p* = 0.009), education (*p* = 0.009), and household income (*p* < 0.001), being higher among participants aged >30 years (25.8% vs. 17.2%), those with pre-tertiary education (31.9% vs. 21.9%), and those with monthly income <10,000 SAR (31.5% vs. 16.3%). Ever receiving FP consultation/services did not vary by age, education, employment, or income (all *p* > 0.05). Barriers to accessing contraception were associated with younger age, unemployment, and lower income (all *p* ≤ 0.001). RH healthcare visit frequency varied by age (*p* = 0.036) and income (*p* < 0.001), while current contraceptive use was associated with age and employment (both *p* < 0.001). Knowledge of age-related fertility decline differed by age (*p* < 0.001), employment (*p* = 0.016), and income (*p* = 0.026), and cultural/family pressure to have more children varied by employment (*p* = 0.020) and income (*p* = 0.013). The ideal number of children differed by age only (*p* < 0.001), with older respondents more frequently preferring 4–5 children (70.9% vs. 55.2%).

**Table 3:** Distribution of key family planning awareness, service use, access barriers, and fertility related attitudes by sociodemographic characteristics (age group, education, employment status, and monthly household income) among participants in Al-baha.

Items	Responses	N (%)	Age			Education			Employment status			Monthly Income		
			≤30	>30	*P* value	Pre-Tertiary	Tertiary	*P* value	Employed	Unemployed	*P* value	≤10K	>10K	*P* value
Awareness of FP initiatives	Yes	210 (23.5%)	40 (17.2%)	170 (25.8%)	**0.009**	46 (31.9%)	164 (21.9%)	**0** **.** **009**	96 (21.1%)	114 (26.0%)	0.086	134 (31.5%)	76 (16.3%)	**<0.001**
	No	682 (76.5%)	192 (82.8%)	490 (74.2%)		98 (68.1%)	584 (78.1%)		358 (78.9%)	324 (74.0%)		292 (68.5%)	390 (83.7%)	
Ever received FP consultation/services	Yes	160 (18.0%)	34 (14.7%)	126 (19.1%)	0.13	26 (18.1%)	134 (17.9%)	0.986	84 (18.5%)	76 (17.4%)	0.654	68 (16.0%)	92 (19.7%)	0.142
	No	732 (82.0%)	198 (85.3%)	534 (80.9%)		118 (81.9%)	614 (82.1%)		370 (81.5%)	362 (82.6%)		358 (84.0%)	374 (80.3%)	
Barriers to accessing contraception	Yes	288 (32.3%)	104 (44.8%)	184 (27.9%)	**<0.001**	48 (33.3%)	240 (32.1%)	0.769	118 (26.0%)	170 (38.8%)	**<0.001**	160 (37.6%)	128 (27.5%)	**0** **.** **001**
	No	604 (67.7%)	128 (55.2%)	476 (72.1%)		96 (66.7%)	508 (67.9%)		336 (74.0%)	268 (61.2%)		266 (62.4%)	338 (72.5%)	
Frequency of RH healthcare visits	Frequently	174 (19.5%)	34 (14.7%)	140 (21.2%)	**0.036**	30 (20.8%)	144 (19.3%)	0.302	78 (17.2%)	96 (21.9%)	0.078	98 (23.0%)	76 (16.3%)	**<0.001**
	Occasionally	246 (27.6%)	60 (25.9%)	186 (28.2%)		46 (31.9%)	200 (26.7%)		120 (26.4%)	126 (28.8%)		142 (33.3%)	104 (22.3%)	
	Rarely	472 (52.9%)	138 (59.5%)	334 (50.6%)		68 (47.2%)	404 (54.0%)		256 (56.4%)	216 (49.3%)		186 (43.7%)	286 (61.4%)	
Current contraceptive use	Yes	370 (41.5%)	40 (17.2%)	330 (50.0%)	**<0.001**	54 (37.5%)	316 (42.2%)	0.29	228 (50.2%)	142 (32.4%)	**<0.001**	168 (39.4%)	202 (43.3%)	0.236
	No	522 (58.5%)	192 (82.8%)	330 (50.0%)		90 (62.5%)	432 (57.8%)		226 (49.8%)	296 (67.6%)		258 (60.6%)	264 (56.7%)	
Knowledge of age-related fertility decline	Extensive knowledge	314 (35.2%)	52 (22.4%)	262 (39.7%)	**<0.001**	44 (30.6%)	270 (36.1%)	0.423	176 (38.8%)	138 (31.5%)	**0** **.** **016**	142 (33.3%)	172 (36.9%)	**0** **.** **026**
	Moderate knowledge	456 (51.1%)	130 (56.0%)	326 (49.4%)		80 (55.6%)	376 (50.3%)		228 (50.2%)	228 (52.1%)		212 (49.8%)	244 (52.4%)	
	Not knowledgeable	122 (13.7%)	50 (21.6%)	72 (10.9%)		20 (13.9%)	102 (13.6%)		50 (11.0%)	72 (16.4%)		72 (16.9%)	50 (10.7%)	
Cultural/family pressure to have more children	Yes	222 (25.0%)	56 (24.1%)	166 (25.2%)	0.759	40 (27.8%)	182 (24.3%)	0.381	98 (21.6%)	124 (28.3%)	**0** **.** **02**	122 (28.6%)	100 (21.5%)	**0** **.** **013**
	No	670 (75.0%)	176 (75.9%)	494 (74.8%)		104 (72.2%)	566 (75.7%)		356 (78.4%)	314 (71.7%)		304 (71.4%)	366 (78.5%)	
Ideal number of children	1–3	212 (24.0%)	86 (37.1%)	126 (19.1%)	**<0.001**	36 (25.0%)	176 (23.5%)	0.313	94 (20.7%)	118 (26.9%)	0.086	100 (23.5%)	112 (24.0%)	0.979
	4–5	596 (67.0%)	128 (55.2%)	468 (70.9%)		90 (62.5%)	506 (67.6%)		314 (69.2%)	282 (64.4%)		286 (67.1%)	310 (66.5%)	
	≥ 6	84 (9.0%)	18 (7.8%)	66 (10.0%)		18 (12.5%)	66 (8.8%)		46 (10.1%)	38 (8.7%)		40 (9.4%)	44 (9.4%)	

Data are presented as *n* (%). For subgroup columns (Age, Education, Employment status, Monthly income), percentages are within each subgroup (column percentages). Age was categorised as ≤30 vs. >30 years. Education was categorised as pre-tertiary vs. tertiary. Employment status was categorised as employed vs. unemployed. Monthly household income was categorised as <10 K vs. ≥10 K SAR. *P* values were derived from Pearson’s *χ*² test (two-sided). Statistical significance was defined as *p* < 0.05. FP, family planning; RH, reproductive health; SAR, Saudi Riyal.

Bold values indicate statistically significant *p*-values (*p* < 0.05).

### 3.9 Sociodemographic and knowledge-related factors associated with current contraceptive use

Age, employment status, awareness of family planning initiatives, and knowledge of age-related fertility decline showed the clearest unadjusted associations with current contraceptive use. In univariable analyses, participants aged >30 years differed significantly from those aged ≤30 years (uOR = 4.80, 95% CI: 3.31–6.97; *p* < 0.001), and employment status was also associated with contraceptive use (uOR = 2.10, 95% CI: 1.60–2.76; *p* < 0.001). Awareness of family planning initiatives in Al-Baha (uOR = 1.39, 95% CI: 1.02–1.89; *p* = 0.039) and knowledge of age-related fertility decline (uOR = 1.35, 95% CI: 1.10–1.65; *p* = 0.004) were additionally associated with current use. In contrast, education, monthly income, prior receipt of family planning consultation/services, barriers to accessing contraception, frequency of reproductive-health visits, cultural or family pressure to have more children, and ideal number of children were not significantly associated with current contraceptive use in unadjusted analyses (Table 4).

**Table 4:** Univariable logistic regression analysis of factors associated with current contraceptive use.

Predictors	uOR	*P*-value	95% CI
Age	4.8	**<0.001**	3.305–6.972
Education	1.219	0.290	0.844–1.760
Employment status	2.103	**<0.001**	1.602–2.760
Monthly Income	1.175	0.236	0.900–1.535
Awareness of FP initiatives in Al-Baha	1.387	**0** **.** **039**	1.016–1.894
Ever received FP consultation/services	0.989	0.948	0.698–1.399
Barriers to accessing contraception	0.929	0.615	0.698–1.236
Frequency of RH healthcare visits	1.059	0.509	0.894–1.255
Knowledge of age-related fertility decline	1.349	**0** **.** **004**	1.101–1.653
Cultural/family pressure to have more children	0.817	0.204	0.599–1.116
Ideal number of children	1.215	0.111	0.956–1.545

Values are unadjusted odds ratios (uOR) derived from separate univariable logistic regression models with current contraceptive use as the dependent variable. Results are presented as uOR with 95% confidence intervals (CI). FP, family planning; RH, reproductive health; SAR, Saudi Riyal.

Bold values indicate statistically significant *p*-values (*p* < 0.05).

**Figure 1:**
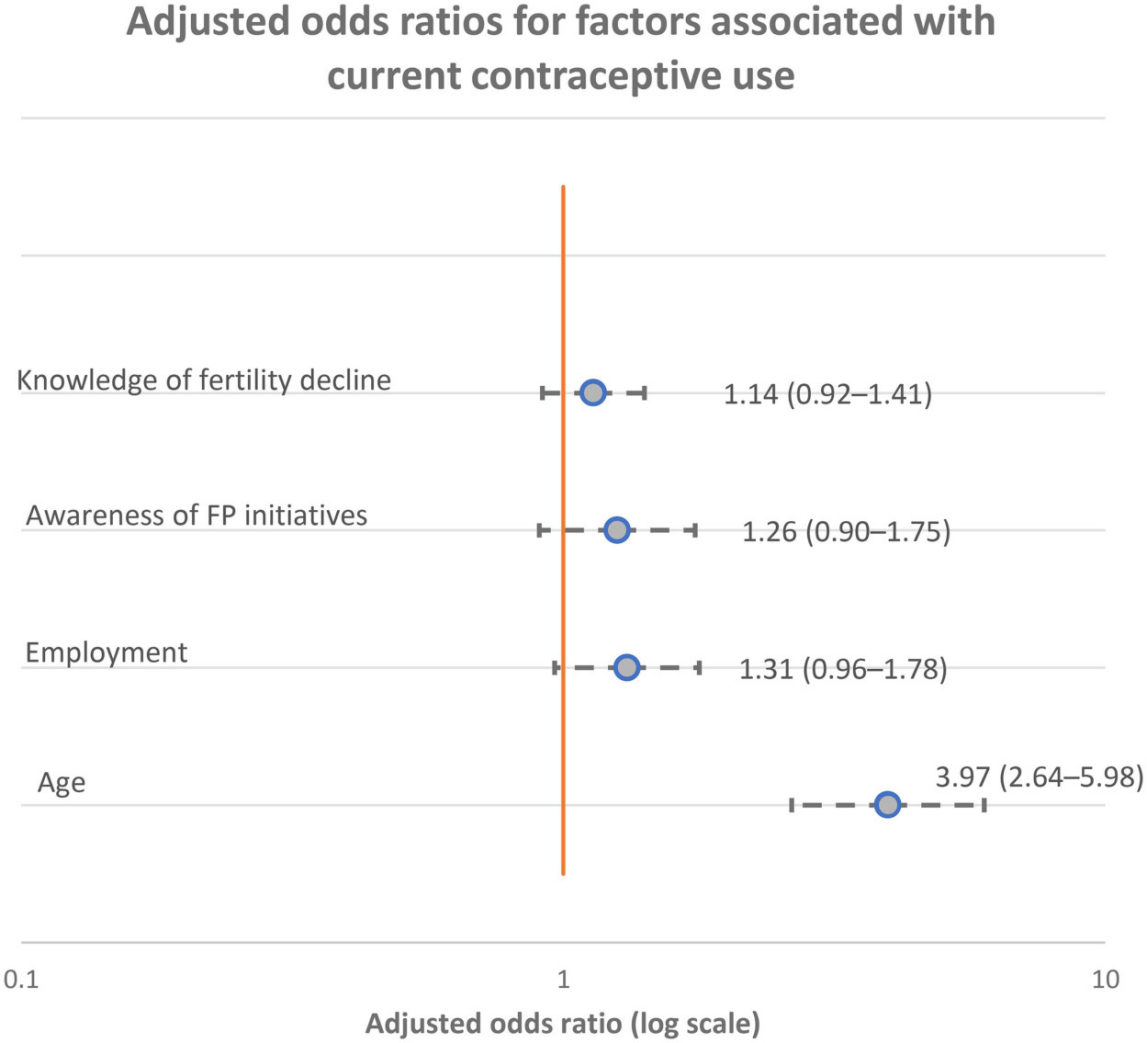
Adjusted odds ratios for factors associated with current contraceptive use. Forest plot showing adjusted odds ratios (aORs) and 95% confidence intervals (CIs) for factors associated with current contraceptive use, estimated using multivariable logistic regression. Dots represent adjusted odds ratios, and horizontal lines indicate 95% confidence intervals. The vertical dashed line denotes an odds ratio of 1.0 (no association). Odds ratios are presented on a logarithmic scale.

In the multivariable logistic regression model, age remained the only independent predictor of current contraceptive use, with participants aged >30 years showing higher odds of current use compared with those aged ≤30 years (aOR = 3.97, 95% CI: 2.64–5.98; *p* < 0.001). After adjustment, employment status (aOR = 1.31, 95% CI: 0.96–1.78; *p* = 0.084), awareness of family planning initiatives (aOR = 1.26, 95% CI: 0.90–1.75; *p* = 0.175), and knowledge of age-related fertility decline (aOR = 1.14, 95% CI: 0.92–1.41; *p* = 0.248) were not statistically significant. Overall, these findings suggest that age was the principal factor independently associated with current contraceptive use in this sample (Figure 1).

## 4 Discussion

This cross-sectional survey identifies a consistent gap between reported geographic access to healthcare and effective engagement with family planning (FP) information and services in Al-Baha. Although most respondents reported being able to reach a healthcare centre within 30 min (94.0%) and many perceived FP tools as available (65.0%), awareness of organised FP initiatives was low (23.5%) and formal counselling/service receipt was limited (18.0%). In parallel, contraceptive non-use remained common (58.5%), and almost one third of respondents reported at least one barrier to accessing contraception (32.3%), most often “other barriers” (13.7%) and limited availability (10.8%). These findings suggest that physical proximity to services is necessary but insufficient; the prevailing challenge appears to involve informational pathways, service integration, and the practical reliability of contraception provision.

Placing these findings in national and global context highlights both relevance and the need for careful denominator interpretation. Recent national statistics in Saudi Arabia report that 29.9% of married women currently use any form of family planning and that 56% of married women of reproductive age have their family planning needs met with modern methods. Earlier national survey reporting also indicated that 32.9% of married women (15–49 years) were using modern contraceptive methods (24, 25).

Direct comparison with the present study should be cautious because this survey sampled adults (≥18 years) via an online convenience approach and measured “current contraceptive use” across respondents rather than restricting to married women of reproductive age. Nonetheless, the combination of moderate reported use (41.5%) alongside very low awareness of initiatives and low counselling uptake indicates that contraceptive behaviour may be occurring with limited structured support and inconsistent contact with provider led FP services. Globally, the World Health Organization reports substantial ongoing need and inequities in access to contraception, emphasising that method availability, misinformation, and service quality remain key barriers even where services exist (4).

A key finding is the mismatch between how people obtain reproductive health information and how often they interact with clinical services. Media was the most reported source of reproductive health information (51.1%), while relatively few cited healthcare sources (18.8%). This aligns with low routine utilisation of reproductive health services: over half reported rare visits (52.9%; once a year or less). In Saudi settings, reliance on social media and the internet for health information is well documented and can be associated with variable quality, misconceptions, and inconsistent health seeking behaviour, potentially undermining informed choice when clinician led counselling is infrequent (26, 27). From a service delivery perspective, limited clinic contact reduces opportunities for routine FP counselling, method review, and management of side effects factors that may reduce continuation and confidence in contraceptive use (28).

The analyses also show that gaps in awareness and barriers are not evenly distributed. Younger participants (≤30 years) were less likely to report awareness of FP initiatives and more likely to report barriers to accessing contraception. Barriers were also more frequently reported by unemployed respondents and those with lower household income, indicating an equity gradient in practical access even when geographic proximity is favourable. These patterns are consistent with broader evidence that lower resource populations experience disparities in reproductive and primary healthcare access and use (5–7, 11, 29). They support shifting from “one size fits all” messaging towards targeted approaches, including youth sensitive counselling pathways that emphasise confidentiality and respectful communication, and service designs that mitigate affordability and availability related constraints.

The multivariable logistic regression further indicates that age was the only factor independently associated with current contraceptive use, with higher adjusted odds among respondents aged >30 years (30, 31). This attenuation of employment, awareness of FP initiatives, and fertility-knowledge effects after adjustment suggests that these factors may operate indirectly through age (e.g., via life-course differences such as parity, accumulated service exposure, and perceived need for birth spacing). 2, 3 In practical terms, the findings imply that observed differences in awareness and reported barriers may not translate into independent differences in contraceptive behaviour once age is accounted for, reinforcing age as a key stratifier for tailoring counselling and outreach.(30, 32).

The study also highlights an educational pattern in which awareness of FP initiatives was higher in the pre-tertiary education group than among those with tertiary education. This directionality differs from what is often expected and may reflect differences in exposure to community-based messaging, variations in media consumption patterns, or selection effects related to online recruitment rather than a true protective effect of lower education. Existing studies in Saudi Arabia suggests that FP awareness and use reflect a complex interplay of information sources, perceived need, and service accessibility rather than education alone (10, 13, 18). This finding warrants cautious interpretation and further investigation using probability based sampling and qualitative methods.

Oral contraceptive pills and intrauterine methods were the most frequently reported contraceptive methods in this survey, and they also represented the largest shares among those currently using contraception. Similar method mix patterns particularly the prominence of oral contraceptives have been reported in Saudi primary care settings, although levels of awareness and utilisation vary by region and study population (10, 13, 18). The observed combination of moderate contraceptive use with low organised counselling and limited awareness of FP initiatives suggests that some contraceptive decision making may occur outside structured FP programmes (for example, through informal advice or self-directed use), reinforcing the need to strengthen consistent, clinician supported counselling, follow up, and method continuation support (18, 28).

Sociocultural influences in this survey appear mixed rather than uniformly restrictive. Most respondents reported that community values do not influence family size decisions and that they did not experience cultural or family pressure to have more children, while a meaningful minority did report pressure, particularly among unemployed and lower income respondents. Evidence from Saudi Arabia indicates that family planning can be framed as acceptable within Islamic perspectives, but local norms and household dynamics can still shape willingness to seek FP services openly and to discuss contraception with clinicians (18, 20, 21, 33). These results support avoiding assumptions of universal sociocultural resistance and instead focusing on subgroup sensitive approaches that address privacy, respectful counselling, and culturally congruent framing.

Health system factors require explicit emphasis, given the low counselling uptake despite favourable geographic access. National family planning guidance emphasises high quality counselling, standard practices, and delivery of FP services across levels of care, particularly primary healthcare, alongside method choice and safe use (34). The current findings suggest several priorities for Al-Baha: strengthening provider capacity for client centred counselling, integrating FP checks into routine primary care encounters, improving continuity for method follow up and side effect management, and strengthening implementation fidelity to service standards, including reliable method availability and clear referral pathways. In addition, given the dominance of media as an information source, digital strategies should be used as a bridge to care, with evidence-based content and clear guidance on when and where to seek clinical counselling, consistent with WHO reporting on persistent service-quality and misinformation barriers (4).

Several limitations should be considered. The online, convenience sampling approach may over represent those who are digitally reachable and more willing to participate, influencing estimates of awareness, information sources, and service use. The cross-sectional design limits causal inference, and self-reported outcomes may be affected by recall and social desirability bias. Nonetheless, this study provides region specific evidence across multiple FP domains and identifies priority groups for targeted interventions, particularly younger adults and socioeconomically disadvantaged respondents, alongside clear system level opportunities to strengthen counselling integration, provider readiness, and policy implementation.

## 5 Conclusion

This cross-sectional survey of adult females in Al-Baha identified a clear gap between reported geographic access to healthcare and engagement with family planning (FP) counselling and related services. Awareness of FP initiatives was low and only a minority reported ever receiving FP consultation/services, while current contraceptive use was reported by fewer than half of respondents and about one third reported at least one access related barrier, most commonly, limited availability. Reproductive health information was most commonly, obtained from media, with fewer participants reporting healthcare sources, and fertility related decision making was frequently influenced by personal preference and household income. These findings support strengthening culturally appropriate health education and community engagement, while also prioritising health system actions particularly provider training, integration of FP counselling within routine primary care, and stronger implementation of service standards (including method availability and referral pathways). Together, these measures may improve informed choice and service quality and align with national health transformation priorities and broader global reproductive health goals.

## Data Availability

The raw data supporting the conclusions of this article will be made available by the authors, without undue reservation.

## References

[1] Lassi ZS Bhutta ZA. Community-based intervention packages for reducing maternal and neonatal morbidity and mortality and improving neonatal outcomes. Cochrane Database Syst Rev. (2015) 2015(3):Cd007754. 10.1002/14651858.CD007754.pub3 25803792 PMC8498021

[2] Miranda J Miller S Alfieri N Lalonde A Ivan-Ortiz E Hanson C Global health systems strengthening: FIGO's strategic view on reducing maternal and newborn mortality worldwide. Int J Gynaecol Obstet. (2024) 165(3):849–59. 10.1002/ijgo.15553 38651311

[3] Coulson J Sharma V Wen H. Understanding the global dynamics of continuing unmet need for family planning and unintended pregnancy. China Popu Dev Stud. (2023) 7(1):1–14. 10.1007/s42379-023-00130-7 PMC10075166 37193368

[4] World_Health_Organization. Family Planning/contraception Methods Geneva. Switzerland: World Health Organization (2025). Available online at: https://www.who.int/news-room/fact-sheets/detail/family-planning-contraception (Accessed February 2, 2026).

[5] Anyatonwu OP San Sebastián M. Rural-urban disparities in postpartum contraceptive use among women in Nigeria: a blinder-oaxaca decomposition analysis. Int J Equity Health. (2022) 21(1):71. 10.1186/s12939-022-01674-9 35581634 PMC9116001

[6] Bozkurt B Planey AM Aijaz M Weinstein JM Cilenti D Shea CM Disparities in maternal health visits between rural and urban communities in the United States, 2016–2018. Perm J. (2024) 28(2):36–46. 10.7812/TPP/23.067 38650474 PMC11232912

[7] Moafa HN van Kuijk SMJ Alqahtani DM Moukhyer ME Haak HR. Disparities between rural and urban areas of the central region of Saudi Arabia in the utilization and time-centeredness of emergency medical services. Int J Environ Res Public Health. (2020) 17(21):7944. 10.3390/ijerph17217944 33138091 PMC7663470

[8] Meo SA Shaikh N Meo AS. The influence of social, demographic and economic factors on fertility trends in Gulf Cooperation Council countries: a longitudinal time trend analysis-1980–2021. Saudi Med J. (2024) 45(9):935–44. 10.15537/smj.2024.45.9.20240437 39218461 PMC11376706

[9] Khraif R Abdul Salam A Al-Mutairi A Elsegaey I. Fertility behaviour of working women in Saudi Arabia: a special case of King Saud University, Riyadh. Hum Fertil (Cambridge, England). (2019) 22(4):246–54. 10.1080/14647273.2018.1449971 29540089

[10] Al Sheeha M. Awareness and use of contraceptives among Saudi women attending primary care centers in al-qassim, Saudi Arabia. Int J Health Sci. (2010) 4(1):11–21. PMC3068803 21475521

[11] Teferi HM Schröders J. Contributing factors for urban-rural inequalities in unmet need for family planning among reproductive-aged women in Ethiopia: a blinder-oaxaca decomposition analysis. BMC women’s Health. (2023) 23(1):158. 10.1186/s12905-023-02304-4 37016342 PMC10074785

[12] Yaya S Uthman OA Ekholuenetale M Bishwajit G. Women empowerment as an enabling factor of contraceptive use in sub-saharan Africa: a multilevel analysis of cross-sectional surveys of 32 countries. Reprod Health. (2018) 15(1):214. 10.1186/s12978-018-0658-5 30572927 PMC6302468

[13] Alselmi A. Family planning unmet need among women attending primary healthcare clinics in Western Region, Saudi Arabia. J Family Med Prim Care. (2023) 12(7):1276–84. 10.4103/jfmpc.jfmpc_1695_22 37649744 PMC10465038

[14] Finlay JE Lee MA. Identifying causal effects of reproductive health improvements on women’s economic empowerment through the population poverty research initiative. Milbank Q. (2018) 96(2):300–22. 10.1111/1468-0009.12326 29870117 PMC5987803

[15] Narula K Kenkre JS Loh WJ Tan T. Obesity, insulin resistance and fertility: unresolved questions and emerging insights. Curr Opin Endocrinol Diabetes Obes. (2025) 32(3):108–14. 10.1097/MED.0000000000000907 40125660

[16] Veras MM Caldini EG Dolhnikoff M Saldiva PH. Air pollution and effects on reproductive-system functions globally with particular emphasis on the Brazilian population. J Toxicol Environ Health Part B, Crit Rev. (2010) 13(1):1–15. 10.1080/10937401003673800 20336577

[17] Wróblewski M Wróblewska W Sobiesiak M. The role of selected elements in oxidative stress protection: key to healthy fertility and reproduction. Int J Mol Sci. (2024) 25(17):9409. 10.3390/ijms25179409 39273356 PMC11395468

[18] Alomair N Alageel S Davies N Bailey JV. Muslim women’s views and experiences of family planning in Saudi Arabia: a qualitative study. BMC Women’s Health. (2023) 23(1):625. 10.1186/s12905-023-02786-2 38007464 PMC10675866

[19] Church AC Ibitoye M Chettri S Casterline JB. Traditional supports and contemporary disrupters of high fertility desires in sub-Saharan Africa: a scoping review. Reprod Health. (2023) 20(1):86. 10.1186/s12978-023-01627-7 37280648 PMC10242605

[20] Schenker JG. Women’s reproductive health: monotheistic religious perspectives. Int J Gynaecol Obstet. (2000) 70(1):77–86. 10.1016/S0020-7292(00)00225-3 10884536

[21] Srikanthan A Reid RL. Religious and cultural influences on contraception. J Obstet Gynaecol Can. (2008) 30(2):129–37. 10.1016/S1701-2163(16)32736-0 18254994

[22] Miskeen E Korkoman S Alhassoun NK Aljuhani RF Alqahtani RAH Alwabari SS Factors influencing family planning decisions in Saudi Arabia. BMC Women’s Health. (2025) 25(1):222. 10.1186/s12905-025-03737-9 40361107 PMC12070546

[23] al-Nasser AN Bamgboye EA. Estimates of fertility levels in a rural community of Saudi Arabia. Int J Fertil. (1992) 37(1):15–8. 1348728

[24] General_Authority_for_Statistics. GASTAT: 66.1% of Births in the Kingdom Occurs in Public Hospitals in 2024. Riyadh, Saudi Arabia: General Authority for Statistics (GASTAT) (2024). Available online at: https://www.stats.gov.sa/en/news?q=birth (Accessed February 2, 2026).

[25] General_Authority_for_Statistics. Household Health Survey 2018. Saudi Arabia: General Authority for Statistics (GASTAT) (2019. (July 28, 2019).

[26] Sumayyia MD Al-Madaney MM Almousawi FH. Health information on social media. Perceptions, attitudes, and practices of patients and their companions. Saudi Med J. (2019) 40(12):1294–8. 10.15537/smj.2019.12.24682 31828284 PMC6969626

[27] AlMuammar SA Noorsaeed AS Alafif RA Kamal YF Daghistani GM. The use of internet and social media for health information and its consequences among the population in Saudi Arabia. Cureus. (2021) 13(9):e18338. 10.7759/cureus.18338 34722089 PMC8551798

[28] Fuentes L Douglas-Hall A Geddes CE Kavanaugh ML. Primary and reproductive healthcare access and use among reproductive aged women and female family planning patients in 3 states. PLoS One. (2023) 18(5):e0285825. 10.1371/journal.pone.0285825 37224157 PMC10208491

[29] Alfaqeeh G Cook EJ Randhawa G Ali N. Access and utilisation of primary health care services comparing urban and rural areas of Riyadh Providence, Kingdom of Saudi Arabia. BMC Health Serv Res. (2017) 17(1):106. 10.1186/s12913-017-1983-z 28153002 PMC5288856

[30] Almalik M Mosleh S Almasarweh I. Are users of modern and traditional contraceptive methods in Jordan different? East Mediterr Health J. (2018) 24(4):377–84. 10.26719/2018.24.4.377 29972232

[31] Mahfouz MS Elmahdy M Ryani MA Abdelmola AO Kariri SA Alhazmi HY Contraceptive use and the associated factors among women of reproductive age in Jazan City, Saudi Arabia: a cross-sectional survey. Int J Environ Res Public Health. (2023) 20(1):843. 10.3390/ijerph20010843 36613165 PMC9820157

[32] Feriani P Yunitasari E Efendi F Krisnana I Ernawati R Tianingrum NA A systematic review of determinants influencing family planning and contraceptive use. Iran J Nurs Midwifery Res. (2024) 29(5):596–607. 10.4103/ijnmr.ijnmr_321_23 39478710 PMC11521132

[33] Alomair N Alageel S Davies N Bailey JV. Factors influencing sexual and reproductive health of Muslim women: a systematic review. Reprod Health. (2020) 17(1):33. 10.1186/s12978-020-0888-1 32138744 PMC7059374

[34] Ministry_of_Health. Family Planning: National Guidelines for Health Care Providers. Evidence-Based Guidance. Riyadh, Saudi Arabia: Ministry of Health (2023).

